# Supplementary figures and images for: Near-infrared spectroscopy outperforms genomics for predicting sugarcane feedstock quality traits
Source: PLoS One. 2021 Mar 4;16(3):e0236853. doi: 10.1371/journal.pone.0236853 (PMC7932073; doi:10.1371/journal.pone.0236853)

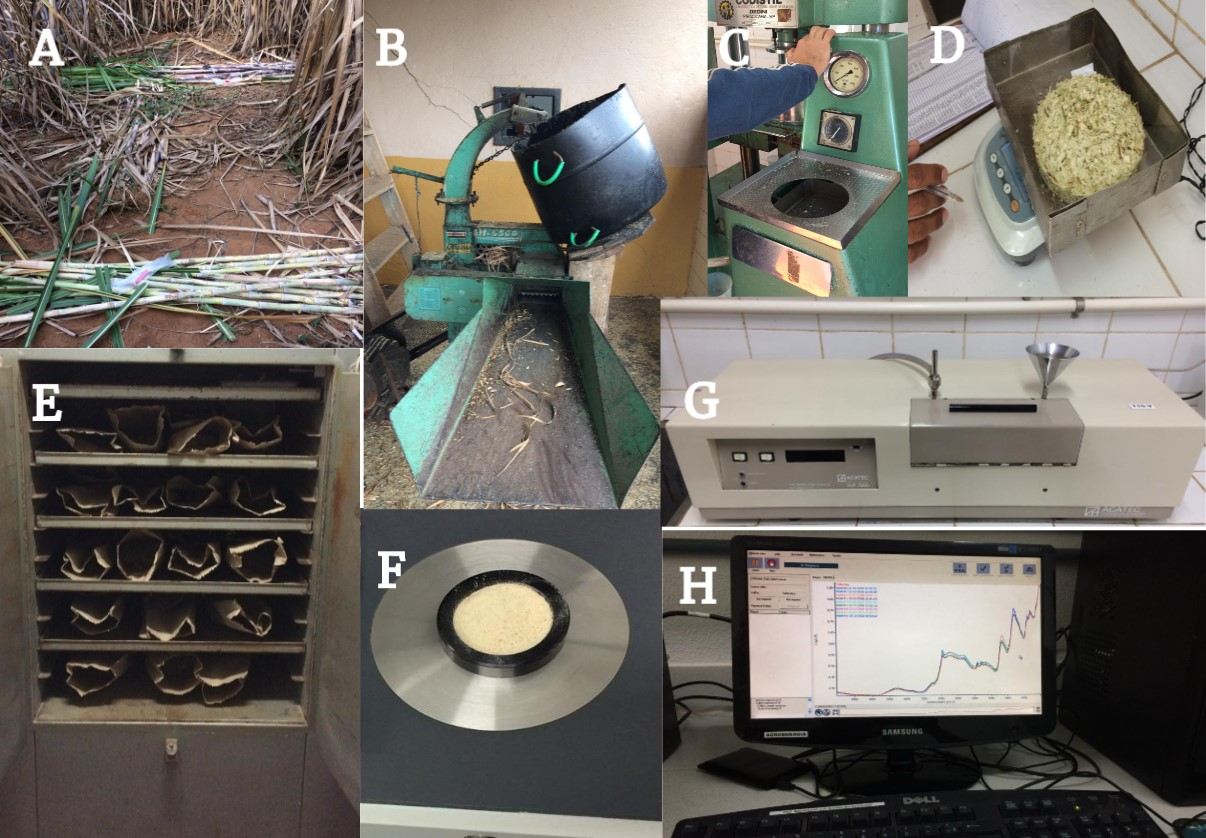

Supplement: S1 Fig — A: stalks being harvested from double-row plots; B: stationary forage chopper machine used to shred stalks; C: hydraulic press used to extract the fiber cake and juice samples; D: fiber cake being weighted; E: samples being dried at a forced-air circulating oven; F: dried ground samples placed onto the NIR instrument window; G: saccharimeter instrument; H: sample spectrum displayed on the computer screen. (TIF) [file pone.0236853.s001.tif]
