# Supplementary material for: Near-infrared spectroscopy outperforms genomics for predicting sugarcane feedstock quality traits
Source: PLoS One. 2021 Mar 4;16(3):e0236853. doi: 10.1371/journal.pone.0236853 (PMC7932073; doi:10.1371/journal.pone.0236853)
Supplement: Attachment — Submitted filename: 14_01_2021_Response_to_reviewers.docx [file pone.0236853.s002.docx]

**Response to Reviewers**

We are grateful for the highly valuable suggestions, comments, and criticizes regarding our manuscript. In the “Revised Manuscript with Track Changes” file, the new inserted texts are highlighted in green, the rearranged paragraphs are highlighted in yellow, and the excluded texts are highlighted in red. The references and changes assigned to lines e.g., L439-490, are referred to the “Revised Manuscript with Track Changes”.

**Editor Comments:**

**1) Provide the keywords for your study;**

Authors: We provided the following keywords in the PLOS ONE Editorial Manager Submission System: NIR, BayesB, PLS regression, single nucleotide polymorphism, plant breeding.

**2) In the Introduction I missed explaining how the qualitative traits of sugarcane can be improved from the tested approaches. Consider providing more details about each of these traits;**

Authors: We further elaborated these points in the introduction (L65-67) and discussion sections (L481-533).

**3) I also feel the need to explain better how NIR can be used for this culture. Are there any previous studies? This needs to be linked to the previous request in the Introduction;**

Authors: We further discussed the point raised by the editor in the discussion section (L455-466).

**4) Apply the Tukey test to compare the strategies (M1, M2 and M3) presented in Figures 4 and 5.**

Authors: This has been addressed in the “Revised Manuscript with Track Changes” (L294-296; L300; L309-310).

**Journal Requirements:**

**1. Please ensure that your manuscript meets PLOS ONE's style requirements, including those for file naming. The PLOS ONE style templates can be found at**

**https://journals.plos.org/plosone/s/file?id=wjVg/PLOSOne_formatting_sample_main_body.pdf and**

**https://journals.plos.org/plosone/s/file?id=ba62/PLOSOne_formatting_sample_title_authors_affiliations.pdf**

Authors: Our manuscript now follows PLOS ONE’s style requirements.

**2. We note that you have stated that you will provide repository information for your data at acceptance. Should your manuscript be accepted for publication, we will hold it until you provide the relevant accession numbers or DOIs necessary to access your data. If you wish to make changes to your Data Availability statement, please describe these changes in your cover letter and we will update your Data Availability statement to reflect the information you provide.**

Authors: We agree with PLOS ONE’s data availability requirement.

**3. Please amend either the title on the online submission form (via Edit Submission) or the title in the manuscript so that they are identical.**

Authors: This has been fixed.

**Reviewer #1:**

**The paper considers prediction of feedstock quality traits in sugar cane using genetic markers (GS) and NIRS. The results show that NIRS outperforms GS and that the combination of NIRS and genetic markers leads to no improvement over use of NIRS alone.**

**(1) Perhaps many readers will consider the result unsurprising because NIRS is much closer to the targeted traits than genetic markers. There are many similar reports in other crops and also with other high-throughput phenotyping data. A more thorough review of this growing literature would be useful.**

Authors: We agree with the reviewer. We have revised the discussion section by adding additional literature (L439-442 and L455-466).

**(2) As the authors state in their introduction, the greatest potential of GS lies in the facility to predict traits even without phenotyping, thus permitting substantial shortening of breeding cycles. By contrast, the samples used for generating the NIRS data are obtained only 26 months after transplanting! Thus, with NIRS you are 26 months slower than GS! Wouldn't most breeders gladly take GS predictions now, even if they are a little less accurate, than waiting another 26 months before NIRS data are in?!**

Authors: The M3 modeling strategy we proposed aimed to increase the predictive ability of sugarcane hybrid clones’ performance for possible release as cultivars. Indeed, from the single perspective of shortening breeding cycles, making such combined prediction could be most useful if spectra could be collected from seedlings or juvenile tissues, around the same time of DNA extraction for sequencing and genotyping; although spectra collection dates may interfere the analysis. In addition, we could postpone NIRS data collection to the first ratoon, 10 to 12 months after planting, but only decided to use 26 months old plants because of the availability of the material at our breeding station. The former approach will be explored in a future work. In the present work, however, our primary goal was simply to investigate whether there is evidence that corroborates our thesis using the currently available dataset. Unfortunately, we did not obtain improvement when combining the two predictors (NIR and SNP). Nevertheless, we further discussed these issues in the manuscript (L417-425).

**(3) The authors use an augmented design to test the 385 clones. They use a two-stage approach, where in the first stage they fit a linear mixed model with random effects for blocks and genotypes and then obtain BLUP of genotype effects to be carried forward to the second state (GS, NIRS-based prediction). There are three issues with this approach that require attention:**

**(i) It is not clear how the replicated checks enter the analysis. If a random effect for genotype is fitted, then it must be assumed that the checks come from the same population as the 385 clones. Is this a realistic assumption?**

Authors: It is indeed a realistic assumption and in our study the replicated checks are well-established cultivars, widely grown throughout the country, and are often used as parents for crosses. Therefore, the population of clones used in our research are somehow related to these checks. The effects of checks were considered random in the mixed model analysis. We modified this paragraph in L132-135 and L208.

**(ii) BLUP are shrinkage estimators. It has been argued by many several authors that these BLUP would need to be unshrunk before submission to the second stage. Animal breeders call these unshrunken estimators deregressed proofs. In animal breeding, use of BLUP is a necessity, essentially because bulls do not give milk and we can only assess their breeding value based on the performance of close female relatives. This is not an issue in plant breeding, where it is perfectly feasible to compute BLUE in the first stage. That this is a prefered approach, is explained, e.g., in Smith, A., B.R. Cullis, and A. Gilmour. 2001. The analysis of crop variety evaluation data in Australia. Aust. N. Z. J.Stat. 43:129–145.**

Authors: We agree with the reviewer. We performed the analysis again using BLUE as suggested by the reviewer. However, the results were similar with BLUP, thus we decided to keep the current results and to show herein the BLUE results:

Fig 1. Correlation of phenotypic values and BLUPs of FIB (top) and PC (bottom).

Results of the analysis using BLUEs into PLS and BayesB models:

Fig 2. Prediction accuracy values for FIB and PC.

Fig 3. Prediction accuracy values for FIB and PC.

**(iii) The BLUE (or BLUP) computed from the augmented design in the first stage are not independent, nor do they usually have constant variance. It is therefore desirable to consider some form of weighting in the second stage. On this point, see Smith et al. (2001) and also Damesa et al. (2019): Comparison of weighted and unweighted stage-wise analysis for genome-wide association studies and genomic selection. Crop Science 59, 2572-2584.**

Authors: We reran the analysis by fitting BLUE first, then extracted residuals and used these residuals as weights in downstream analysis. However, the residuals we obtained were very small (near zero) for all observations, possibly because there were small block effects either. Consequently, the weighted two-stage analysis resulted in poor accuracies of prediction. We used an augmented block design and observed a very small variance due to the block effect, which explains the high correlation between raw phenotypes and adjusted means (BLUEs). In contrast, Damesa et al. (2019) used an alfa-lattice design, which allows a better estimation of genotype means. Also, the authors observed minor advantages when comparing weighted and unweighted approaches and stated that the unweighted method is acceptable for most practical purposes.

**(4) The authors use "random sampling cross-validation" (L247-248). Why not use k-fold cross-validation, as is much more common for these kinds of analysis?**

Authors: The repeated random sampling CV is also common in plant breeding and has been used in many papers. For example, <https://www.g3journal.org/content/2/12/1595.long> and other cited references. We believe the choice of CV is of less concern as long as we are consistently applying the same CV scheme throughout the paper.

**(5) The authors correlate univariate BLUPs of the two target traits. What does this really estimate? I think it would be much better to fit a bivariate model and obtain a REML estimate of the genetic correlation.**

Authors: We fitted a bivariate GBLUP to estimate genetic correlation between the two traits as suggested by the reviewer. However, we found similar results compared to the correlation using univariate BLUPs. Since there was no difference, we decided to keep it as is (L269-272).
**(6) The results report a "prediction accuracy" (L277) but I think this criterion was never explained in M&M.**

Authors: The criterion can be found in line 259-260.

**(7) A more gender-neutral translation of UAV is "uncrewed aerial vehicle", rather than "unmanned aerial vehicle". I am not insisting on this point, but am sure a lot of readers would appreciate this usage.**

Authors: This change has been made in the manuscript (L84).

**Reviewer #2:**

**The manuscript addresses an important topic regarding the incorporation of new tools in plant breeding, aiming to improve accuracy, response to selection, and reduce time and costs. However, there are two main issues to be solved:**

**The authors concluded that NIR outperforms GS. Regarding accuracy, it can be real. However, it is not a fair and complete comparison between these tools. The former does not allow to reduce the cycle between generations. On the other hand, via GS, it is possible to speed up the breeding process. Therefore, I suggest include comparisons in terms of Response to Selection, dividing the accuracy by time (of each method).**

Authors: We modified the discussion section and provided a more thorough review of these points, in order to meet inconsistencies (L495-533).

**Another concern is the GS model. The authors did not consider the dosage for each locus. Thus, all classes of heterozygous individuals are sum up in just one, reducing the resolution of the GS and estimation of the models. Furthermore, they did not consider dominance to predict hybrid clones, where heterosis is significant. I suggest both corrections.**

Authors: The implementation of genomic prediction in sugarcane has been so far only a proof of concept. The consideration of allele dosage is not trivial, and methods able to accommodate allele dosage are still not effective, especially for complex poly-aneuploidy organisms such as sugarcane. We revised and elaborated the discussion section on this topic. (L401-416).

In regard to the consideration of non-additive effects, we modified our discussion. Although there is clear evidence that suggests the improvement of non-additive effects to predict clones’ performance, we believe that models that only account for additive effects can still be useful (L394-400). However, this approach will be explored in a forthcoming work.
